# Cerebral blood flow evaluation of intensive rosuvastatin therapy in stroke/transient ischemic attack patients with intracranial arterial atherosclerotic stenosis study: Rationale and design

**DOI:** 10.1002/brb3.689

**Published:** 2017-04-28

**Authors:** Xuting Zhang, Ying Zhou, Sheng Zhang, Wenhong Ding, Min Lou

**Affiliations:** ^1^ Department of NeurologySchool of MedicineThe Second Affiliated Hospital of Zhejiang UniversityHangzhouChina; ^2^ Department of RadiologySchool of MedicineThe Second Affiliated Hospital of Zhejiang UniversityHangzhouChina

**Keywords:** cerebral hemodynamics, cerebral perfusion, clinical trial, intracranial atherosclerotic stenosis, ischemic stroke, statins

## Abstract

**Introduction:**

The risk of recurrent stroke is high in patients with intracranial atherosclerotic stenosis (ICAS). Statin use has been demonstrated to decrease the incidence of stroke by reducing atherosclerotic plaque burden. However, its effect on the hemodynamic situation and cerebral perfusion status has not yet been validated. With the use of computed tomography perfusion (CTP), we aim to evaluate the impact of Rosuvastatin on cerebral hemodynamic changes, as well as the downstream perfusion.

**Method:**

Cerebral bood flow evaluation of intensive rosuvastatin therapy in patients with intracranial arterial atherosclerotic stenosis (CEIRIS) is a single‐center, prospective, randomized, parallel‐group, and open‐label trial, and it will include 50 participants as estimated. Patients with moderate to severe (50%–99%) ICAS are randomized 1:1 to 10 mg/day or 20 mg/day Rosuvastatin and followed every 13 weeks until 52 weeks. The primary outcome for the trial is the change in the relative regional cerebral blood flow evaluated by CTP after 52 weeks of Rosuvastatin treatment. The secondary outcomes are cerebral blood volume, change in the degree of stenosis of the target vessel and lipid parameters.

**Conclusion:**

The CEIRIS trial about the effects of statin on the temporal hemodynamic progression of ICAS may extend our understanding of the basic pathophysiology and mechanisms of stroke in ICAS patients.

## 1 Introduction

Intracranial atherosclerotic stenosis (ICAS) accounts for 30%–50% stroke or transient ischemic attack (TIA) in Asian (Liu, Wang, Wong, & Wang, 2011), and is highly associated with the risk of stroke recurrence. The risk of recurrent stroke in the territory of symptomatic stenotic artery s as high as 23% during the first year in patients with severe stenosis (defined as 70% or more luminal narrowing), while the annual risk of moderate stenosis (50%–69%) is approximately 10% (Kasner et al., 2006).

### 1.1 Hemodynamic compromise in ICAS

Hemodynamic compromise is one of the possible mechanisms of cerebral infarction secondary to ICAS, especially in the dista region of stenosis. Intracranial atherosclerosis may lead to downstream ischemia in a specific arterial territory because of hypoperfusion. Under normal condition, autoregulatory cerebral vasodilatation occurs and increases the corresponding blood flow to maintain the cerebral blood flow (CBF) against the effect of hypoperfusion. When vasomotor compensatory mechanisms fail to impair the hemodynamics, the stenosis may become symptomatic because of cerebral perfusion insufficiency (Markus & Cullinane, 2001). On the other hand, the low CBF velocity confers the risk of ischemic stroke by reducing the washout of small emboli (Caplan & Hennerici, 1998). Thus, hemodynamic significance of ICAS may also yield a good predictor for stroke risk, leaving it important to identify the reduction in the cerebral perfusion pressure. With transcranial doppler and 2D phase‐contrast MR imaging, measuring hemodynamic changes can classify the severity of stenosis, predict the risk of recurrent stroke, and detect in‐stent restenosis. (Aminhanjani et al., 2010; Amin‐Hanjani et al., 2010; Baumgartner, Mattle, & Schroth, 1999; Wang, Xing, Li, Han, & Chen, 2014). However, hemodynamic changes in the intracranial steosis induced by rosuvastatin is not well‐characterized, particularly when measured with computed tomography perfusion (CTP).

### 1.2 Hemodynamic assessment with CTP

Computed tomography perfusion is a widely available diagnostic tool that provides quick and minimally invasive assessment of cerebral perfusion (Bash et al., 2005). It can be used to identify the ischemic lesions and regional hypoperfusion. The parameters of CTP include CBF, cerebral blood volume (CBV), and mean transit time (MTT). CBF stands for the flow rate of the volume of blood through cerebral vessel. Relative regional CBF (rCBF) is evaluated as the percentage of radioisotope counts in the region of interest (ROI) of the affected side against the corresponding ROI of the unaffected contralateral side. CBV is the volume of flowing blood. MTT indicates the time required for blood to pass through the tissue.

### 1.3 Rationale for the CEIRIS study

Alternative therapies are urgently needed for patients with ICAS. Statin, 3‐hydroxy‐3‐methylglutaryl coenzyme, which is a reductase inhibitor, can decrease the incidence of TIA or ischemic stroke and improve the stroke outcome (Prabhakaran & Romano, 2012; Tan, Kuo, Lin, & Chen, 2009). There are many potential mechanisms through which statin may reduce the stroke risk, including stabilization of atherosclerostic plaques and effection on thrombosis pathways (Amarenco, Labreuche, Lavallee, & Touboul, 2004; Crouse et al., 2007; de Groot et al., 1998; Salonen et al., 1995). Few studies focus on the relationship between statin therapy and cerebral perfusion. Several animal models and clinical trials suggested that statin administration augmented the CBF and improved cerebral vasomotor reactivity by increasing the NO bioavailability (Giannopoulos, Katsanos, Tsivgoulis, & Marshall, 2012). For example, simvastatin upregulates endothelium nitric oxidesynthase, which is associated with increased basal hemispheric CBF and protection against ischemic injury in normocholesterolemic mice (Endres et al., 1998). In Zhou's study, patients with ICAS treated with intensive atorvastatin therapy (40 mg/day) gained significant improvement compared with standard dose therapy (20 mg/day) in the percentage of stenosis on computed tomography angiography (CTA) and in the perfusion of downstream territory measured with CTP (Zhou et al., 2014).

However, whether intensive Rosuvastatin therapy, compared with standard Rosuvastatin therapy can improve hemodynamic situation and cerebral perfusion status in patients with ICAS has not been illustrated. Based on those studies, we hypothesize that intensive Rosuvastatin may alleviate the symptoms of ICAS not only through enhancing the stability of atherosclerotic plaques, but also by changing the hemodynamic status around the plaque and increasing the cerebral flow in the downstream territory. Therefore, we design this study to analyze the impact of Rosuvastatin on the hemodynamic changes as well as the downstream perfusion which is evaluated by the CTP. Since statin is recommended by AHA/ASA guidelines to reduce the risk of stroke presumed to be of atherosclerotic origin, we set a standard dose Rosuvastatin arm instead of a placebo arm.

## 2 Methods

### 2.1 Design

**Figure 1:**
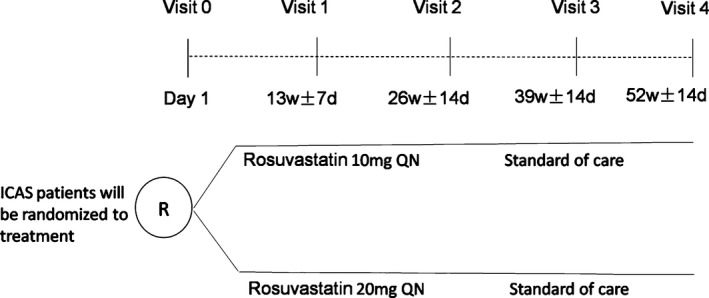
The CEIRIS study design. ICAS, intracranial atherosclerotic stenosis; QN, every night; R, randomization; w, weeks; d, day (s)

This study is a single‐center, prospective, randomized, parallel‐group, open‐label study. Chinese patients with ICAS with 50%–99% stenosis are randomized 1:1 to 10 mg/day or 20 mg/day Rosuvastatin and followed for 52 weeks on study drug (Figure 1). It is designed to validate the hypothesis that the treatment of Rosuvastatin (20 mg/day for 52 weeks) could improve the cerebral perfusion compared with standard treatment. The study protocol conforms to the ethical guidelines of the 1975 Declaration of Helsinki approval by the institution's human research committee of the second affiliated hospital of Zhejiang university, School of medicine. Written informed consent will be obtained from all patients. This study has been registered in ClinicalTrials.gov (NCT02594800).

### 2.2 Participants eligibility

An estimated 50 subjects will be enrolled in our department. The patient population comprises men and women aged 18–80 years who can be randomized within 3 months of an acute ischemic stroke or TIA of the following conditions (Table 1): unilateral intracranial internal carotid artery/middle cerebral artery M1/basilar artery stenosis between 50% and 99%; statin naïve, which is defined as receiving no statin therapy within 3 months. MRA is used to assess the degree of stenosis. The degree of stenosis is evaluated according to the published method in the Warfarin‐Aspirin Symtomatic Intracranial Disease Study (Samuels, Joseph, Lynn, Smith, & Chimowitz, 2000).

**Table 1:** Inclusion criteria

1. Provision of informed consent before any study‐specific procedures
2. Male and female adults aged 18–80 years old
3. Recent (within 3 months) ischemic stroke or transient ischemic attack
4. Unilateral intracranial internal carotid artery/middle cerebral artery M1/basilar artery symptomatic stenosis between 50% and 99%
5. Statin naïve: defined as receiving no statin therapy within 3 months

Those patients with the presence of any of cardiac sources of embolism or hemorrhagic infarction are excluded. Trial of Org 10172 in Acute Stroke Treatment classification is used to determine the cardioembolism etiology of ischemic stroke (Jr et al., 1993). In addition, patients with other major cerebral arteries: extracranial or intracranial internal carotid artery/middle cerebral artery M1/basilar artery/extracranial or intracranial vertebral artery (>50%) ipsilateral or contralateral to the symptomatic intracranial stenosis are not eligible for participation. The full list of exclusion criteria is provided in Table 2.

**Table 2:** Exclusion criteria

1. Any hemorrhagic stroke
2. Presence of any of cardiac sources of embolism
3. Stenosis of other major cerebral arteries (extracranial or intracranial internal carotid artery/middle cerebral artery M1/basilar artery/extracranial or intracranial vertebral artery >50%)
4. Nonatherosclerotic vasculopathy, such as dissection, vasculitis, vasospasm, dissecting aneurysm, radiation‐induced vasculopathy, or MoyaMoya disease, etc.
5. Previous treatment of target lesion with a stent, angioplasty, or other mechanical device, or plan to perform staged angioplasty within 2 years
6. Having severe neurological deficits that render the patient incapable of living independently
7. Life expectancy of patients is less than 2 years
8. The patient has a known or suspected allergy to the study medication(s) or the class of study medication to be administered
9. The patient has to take medicines as follows: Hormonal therapy, Cyclosporine, and other lipid‐lowering agents: fish oil, Fibrates, niacin, Probucol, etc.
10. The patient has any other clinically significant medical condition that, in the opinion of the investigator, could impact the patient's ability to successfully complete the trial
11. The patient has ALT or AST >3 times the upper limit of normal, serum creatinine >2.0 mg/dl, GFR <30 ml/min or has abnormal laboratory values which are deemed clinically significant by the investigator
12. Pregnancy, lactation, and women who are potential to become pregnant but are not willing to take any actions for contraception

### 2.3 Randomization

Patients who meet all of the inclusion and none of the exclusion criteria will be randomly assigned to receive standard dose (10 mg/day) or intensive‐dose (20 mg/day) Rosuvastatin in a 1:1 ratio. Randomization occurs on the day of entering into the study. A randomization list will be generated using a computerized procedure and subjects will be randomized strictly sequentially as they are eligible for randomization.

### 2.4 Intervention

At visit 0 (randomization), eligible patients are randomly assigned to one of the two treatments:

- Treatment 1: Rosuvastatin 10 mg per night (one tablet).
- Treatment 2: Rosuvastatin 20 mg per night (two tablets).

Patients are treated with standard‐of‐care therapy at the discretion of the investigator and followed every 13 weeks until 52 weeks.

### 2.5 Primary outcome

The primary outcome for the trial is the change in the relative rCBF evaluated by the CTP after 52 weeks of Rosuvastatin treatment. ROI will be studied on global hemisphere.

### 2.6 Secondary outcomes

The secondary outcome is the change in the CBV after 52 weeks treatment. Other secondary endpoints include change in the degree of stenosis of the target vessel and lipid parameters, such as low density lipoprotein cholesterol, high density lipoprotein cholesterol, total cholesterol, apolipoprotein A, and apolipoprotein B.

Safety endpoints include the effect of Rosuvastatin on myopathic events, hepatic function, renal function, and other adverse events. Myopathic events refer to elevated creatine kinase level to five times of the normal range. Abnormal liver function is defined as glutamic‐pyruvic transaminase or glutamic‐oxaloacetic transaminase is up to three times of the normal range. Renal insufficiency is defined as an estimated glomerular filtration rate <60 ml/min/1.73 m using the Chronic Kidney Disease Epidemiology Collaboration equation.

### 2.7 Assessment of outcomes and follow‐up

All patients will have contacts at 13 weeks (±7 days), 26 weeks (±14 days), 39 weeks (±14 days) and 52 weeks (±14 days) after randomization. At each contact, lab tests including liver function, renal function, and lipid paremeters will be completed. For Visit 2 (26 weeks ± 14 days) and Visit 4 (52 weeks± 30 days), patients will be re‐evaluated with CTP. CTP assessor is blinded to the treatment which the patient is receiving. Considering collateral circulation in acute stroke may affect the cerebral haemodynamics, we will evaluate the collateral status with CTP using the methods reported by Menon BK (Menon et al., 2011) and analysis this factor in the final statistical analyses. The education and counseling about the symptoms and signs of myalgia, liver dysfunction, and renal insufficiency will be repeated and concomitant medications (including antihypertension medication and cilostazol) will be collected. Adverse events/serious adverse events will be collected and recorded in the case report form from the signing of informed consent throughout the study until the last visit.

### 2.8 Sample size and statistical analyses

According to the previous study (Zhou et al., 2014), 0.15 ml/100 g/min of rCBF change was observed in intensive‐dose atorvastatin therapy (IAT, 40 mg/day, *n* = 37), compared with 0.07 ml/100 g/min on low‐dose atorvastatin therapy (LAT, 10 mg/day, *n* = 38) and 0.09 ml/100 g/min using standard dose atorvastatin therapy (SAT, 20 mg/day, *n* = 37). The standard deviations of rCBF change as baseline in each group was derived using pooled standard deviations of rCBF at baseline and the 52 week. To be conservative, the larger standard deviation (*SD*) of comparing Rosuvastatin 20 mg and Rosuvastatin 10 mg is used for the estimation of sample size. In this study, it is assumed that Rosuvastatin 20 mg will have similar effect as with IAT and Rosuvastatin 10 mg as with LAT or SAT.

Based on these information, a sample size of 22 patients per group at a 1:1 allocation ratio could have an 80% power to detect an estimated difference of 0.07 between (Rosuvastatin 20 mg) and (Rosuvastatin 10 mg) in rCBF (primary outcome) after 52‐week treatment, assuming a standard deviation of 0.08 and at a two‐sided significance level of 0.05. Taking into account an estimated 10% rate of non‐assessable patients, a total of 50 patients will have to be randomized in the study.

All enrolled and randomized patients will be included in the full analysis set. Patients who withdraw consent to participate in the study will be included up to the date of their study termination. Subjects will be analyzed with a modified intention‐to‐treat approach. We will use ANOVA test to calculate the significance of the primary and secondary end point. Categorical variables will be described by counts and proportions, and continuous variables will be described by mean, *SD*, median, and interquartile range. A two‐sided level of significance of 0.05 will be applied to general comparison.

## 3 Discussion

Prevention of recurrent stroke in ICAS patients remains an important issue in China, since in the Chinese Intracranial Atherosclerosis Study, recurrent stroke occurred in 3.82% for patients with 50%–69% stenosis and in 5.16% for those with 70%–99% stenosis (Wang, Zhao, et al., 2014). In SAMMPRIS trial, aggressive medical management containing intensive lipid‐lowering therapy was superior to percutaneous transluminal angioplasty and stenting for intracranial artery stenosis (Derdeyn et al., 2014). There are various potential mechanisms responsible for statin‐associated stroke prevention, including various effects on thrombotic pathways and plaque stabilization, since more regressed, stationary or less progressed ICAS was found in the statin group (Tan et al., 2009).

Few studies, however, have empirically explored the underlying hemodynamic effect of statin in ICAS. This study has been the first investigator‐initiated, prospective, randomized, and open‐label study that was designed to evaluate the effect of different doses of Rosuvastatin treatment on the perfusion status in patients with ICAS. Our trial will evaluate whether intensive Rosuvastatin is safe and effective in improving cerebral hemodynamic status of ICAS. With a high standard of trial design and carefully monitored site, the results of this study will produce reliable data evaluating the relationship between the increased perfusion and Rosuvastatin treatment in patients with ICAS.

Current medical therapies for ischemic stroke are directed primarily at reducing the risk of thromboembolism (antithrombotic agents) and stabilizing plaque (statins), but do not address an underlying state of hypoperfusion. The data regarding the effects of statin on the temporal hemodynamic progression of ICAS may extend our understanding of the basic pathophysiology and mechanisms of stroke in ICAS patients. The results may provide an exploratory mechanism of reducing the risk of ischemic stroke by improving hemodynamic status.

### 3.1 Potential study limitation

Collateral circulation may alter cerebral hemodynamics in acute stroke. Though we can use the CTP‐based dynamic CTA to evaluate the collateral status in anterior circulation, and adjust this factor in the final analysis, it is still difficult to assess the collateral circulation in posterior circulation.

## Conflict of Interest

The authors have no conflicts of interest to disclose.
